# Outcomes in rheumatoid arthritis patients treated with abatacept: a UK multi-centre observational study

**DOI:** 10.1186/s41927-020-00173-0

**Published:** 2021-02-04

**Authors:** Ernest Choy, Lara Groves, Daniel Sugrue, Michael Hurst, John Houghton, Srinivasan Venkatachalam, Yusuf I. Patel, James R. Maxwell, Kevin G. Pollock, Sadie Henning

**Affiliations:** 1 CREATE Centre, Division of Infection and Immunity, Cardiff University School of Medicine, Wales, UK; 2 Cardiff and Vale University Health Board, Cardiff, Wales, UK; 3 Health Economics and Outcomes Research Ltd, Cardiff, UK; 4 Cannock and Wolverhampton Rheumatology Centre, The Royal Wolverhampton NHS Trust, Wolverhampton, UK; 5 Hull University Teaching Hospitals NHS Trust, Hull, UK; 6 Sheffield Teaching Hospitals, Sheffield, UK; 7 Bristol Myers Squibb, Uxbridge Business Park, Sanderson Road, Uxbridge, Middlesex UK

**Keywords:** Abatacept, Biologic, DMARD, Disease activity, Observational study, Rheumatoid arthritis, Time on treatment

## Abstract

**Background:**

Rheumatoid arthritis (RA) is an inflammatory autoimmune disease that causes chronic synovitis, resulting in progressive joint destruction and functional disability and affects approximately 400,000 people in the UK. This real-world study aimed to describe the characteristics, treatment patterns and clinical outcomes of patients who received abatacept in UK clinical practice.

**Methods:**

This was a multi-centre, retrospective, observational study of patients with RA treated with abatacept at four UK centres between 01 January 2013 and 31 December 2017. Data were collected from medical records of each patient from the index date (date of first bDMARD initiation) until the most recent visit, death or end of study (31 December 2017).

**Results:**

In total, 213 patients were included in the study. Patients received up to eight lines of therapy (LOTs). Treatment with abatacept, or any other bDMARD, was associated with reductions in DAS28-ESR and DAS28-CRP scores at 6 and 12 months. The distribution of EULAR responses (good/moderate/no response) tended to be more favourable for patients when receiving abatacept than when receiving other bDMARDs (22.8%/41.3%/35.9% versus 16.6%/41.4%/42.1% at 6 months, and 27.9%/36.1%/36.1% versus 21.2%/34.5%/44.2% at 12 months). Patients receiving abatacept at LOT1 (*n* = 68) spent significantly longer on treatment compared with patients receiving other bDMARDs (53.4 vs. 17.4 months; *p*< 0.01); a similar trend was observed for LOT2. Among patients who discontinued after 6 months, a greater proportion experienced infection requiring antibiotics when receiving other bDMARDs compared to those receiving abatacept.

**Conclusions:**

RA patients who received bDMARDs, including abatacept, experienced reduced disease activity. When receiving abatacept as first or second line of therapy, patients persisted with treatment significantly longer than those receiving other bDMARDs.

**Supplementary Information:**

The online version contains supplementary material available at 10.1186/s41927-020-00173-0.

## Background

Rheumatoid arthritis (RA) is a systemic autoimmune disease driven by both pro-inflammatory cytokines and pathogenic autoantibodies that causes chronic synovitis, resulting in progressive joint destruction and functional disability [1–5]. RA-driven inflammatory processes are also associated with interstitial lung disease and cardiovascular disease, leading to increased disability and mortality [1]. It is estimated that over 400,000 people in the UK have RA [6, 7], and this progressive and often debilitating disease can have a detrimental effect on quality of life for patients, their families, and carers [8, 9].

Whilst there is no cure for RA, current therapies aim to slow disease progression by reducing inflammation and minimising joint damage. National and international treatment guidelines recommend treatment with biological DMARDs (bDMARDs), such as abatacept, after failure of conventional synthetic disease-modifying anti-rheumatic drugs (csDMARDs) when the treatment target is not achieved or poor prognostic factors are present [10–12]. Positivity for rheumatoid factor (RF) and/or anti-citrullinated protein-peptide antibodies (ACPA) is a useful diagnostic and prognostic marker for RA as they may affect treatment response, with some evidence that the presence of these autoantibodies is associated with poorer outcomes [13–18].

International observational studies demonstrate the impact of abatacept on patient outcomes [19], as well as prognostic factors for abatacept retention [20] and durability [21, 22]. Others have found that abatacept performs favourably in terms of treatment persistence when compared to tumour necrosis factor inhibitors (TNFi) [23] and other non-TNFi [24]. However, clinical effectiveness and treatment patterns of abatacept for treating RA have not been well-studied in real-world routine practice, notably in a UK population. Therefore, this study aimed to investigate the characteristics of patients who received abatacept in the UK real-world setting, their treatment patterns and disease activity.

## Methods

This study was a multi-centre, retrospective, observational chart review of patients with RA treated with abatacept at four UK centres: University Hospital of Wales, The Royal Wolverhampton NHS Trust, Hull University Teaching Hospitals NHS Trust, Sheffield Teaching Hospitals NHS Foundation Trust. The research window was from 01 January 2013 to 31 December 2017. The index date was defined as the date of first bDMARD initiation (irrespective of line of therapy (LOT)). Data were collected for each patient until the most recent visit (up until 31 December 2017) or death.

The study received ethical approval form the Yorkshire & The Humber - South Yorkshire Research Ethics Committee (Reference: 18/YH/0412) and research permissions from the Health Research Authority (Reference: 242712).

Eligible patients were identified through screening of medical records at the study centres. Patients were included in the study if they met all the following criteria: aged ≥18 years on index date (date of first bDMARD initiation); received abatacept for the treatment of RA at any LOT within the research window (01 January 2013 to 31 December 2017); medical records contained at least two of disease activity score for 28 joints - erythrocyte sedimentation rate (DAS28-ESR) or disease activity score for 28 joints – C-reactive protein (DAS28-CRP) scores as part of RA treatment monitoring: one on index date and another score at 6 ± 3 months and/or 12 ± 3 months following treatment initiation. Patients with preclinical RA, including undifferentiated arthritis, and/or with comorbid RA or other types of (non-rheumatoid) arthritis were excluded from the study.

A bespoke electronic case report form (eCRF) was used to record all study data. Data from each eligible patient were extracted from medical records and entered into the eCRF by the investigator or their assigned staff at each study centre. The eCRF allowed investigators to remove patient identifiers and ensure only pseudo-anonymised patient-level data were analysed. Built-in validation checks were also used to facilitate accurate and valid data entry. Data collection was entirely retrospective and did not involve any direct patient contact.

Baseline patient demographic and clinical characteristics were analysed descriptively, where baseline was defined as the index date (date of first bDMARD initiation). Summary statistics (mean, standard deviation, median and interquartile range [IQR]) were presented for continuous variables, with counts, proportions, and percentages presented for categorical variables. Analyses were also stratified by positive and/or negative ACPA and RF status. Time on treatment was summarised as a continuous variable and time-to-event curves (based on time to treatment discontinuation or end of follow up [EOFU]) were derived using the Kaplan-Meier method. Log-rank and Wilcoxon tests were used to ascertain statistically significant differences in time on treatment between subgroups. Changes in DAS28 scores were calculated from LOT initiation: 6 month scores were calculated using the recorded score closest to the end of month 6 and included scores recorded between months 4 and 9; 12 months scores were calculated using the recorded score closest to the end of month 12 and included those recorded between months 10 and 15. Associations between change in DAS28 (ESR or CRP) score and bDMARD were modelled using a linear mixed-effects model.

## Results

In total, 213 patients met eligibility criteria and were included in the study. The mean age of patients was 55.2 years and 71.4% of patients were female. The majority of patients (70.4%) were diagnosed with RA prior to 2009 and the median (IQR) RA disease duration was 3.9 (2.1–8.5) years (Table 1). The study population contained: ACPA+/RF+, *n* = 76; ACPA+/RF-, *n* = 7; ACPA−/RF+ *n* =10; ACPA−/RF-, *n* = 22; unknown RF/ACPA status, *n*=98 (Table 1). Patients’ demographic and clinical characteristics were also broadly comparable when stratified by LOT and treatment received.

**Table 1:** Patient demographics, clinical characteristics and disease activity scores at baseline

Variable	All patients (***n*** = 213)	By ACPA/RF status				
		ACPA+/RF+ (***n*** = 76)	ACPA+ /RF- (***n*** = 7)	ACPA- /RF+ (***n*** = 10)	ACPA−/RF- (***n*** = 22)	ACPA and/or RF status not recorded (***n*** = 98)
**Demographics (at index date unless specified)**						
**Age (years old)**						
Mean (SD)	55.2 (13.1)	56.1 (12.9)	46.2 (6.6)	54.6 (13.3)	54.0 (15.2)	55.5 (13.1)
Median (IQR)	55.4 (46.9–64.6)	56.2 (48.3–64.1)	47.8 (43.9–48.8)	51.4 (45.1–63.5)	57.5 (43.6–67.0)	55.1 (48.0–64.7)
**Sex**						
Female, n (%)	152 (71.4%)	54 (71.1%)	^a^	10 (100.0%)	17 (77.3%)	66 (67.3%)
**Weight (kg)**						
Mean (SD)	79.4 (17.8)	79.7 (18.7)	80.1 (12.6)	78.7 (16.0)	90.2 (23.0)	77.3 (16.1)
Median (IQR)	77.2 (65.8–89.7)	76.2 (65.6–95.0)	79.4 (70.1–89.4)	71.9 (68.8–84.9)	86.2 (75.0–91.0)	76.8 (64.9–88.3)
**SBP (mmHg)**						
Mean (SD)	134.8 (19.3)	131.5 (21.2)	129.7 (14.6)	145.3 (24.7)	143.8 (15.4)	135.0 (17.1)
Median (IQR)	133.0 (120.0–146.5)	129.0 (114.5–142.5)	136.0 (124.5–138.0)	144.0 (124.8–163.5)	139.0 (134.0–158.5)	133.0 (120.0–147.5)
**DBP (mmHg)**						
Mean (SD)	78.7 (10.9)	77.2 (11.0)	70.7 (7.4)	73.1 (11.8)	85.1 (12.1)	80.0 (10.0)
Median (IQR)	78.0 (70.0–86.0)	75.0 (70.0–84.5)	68.0 (66.5–73.5)	71.0 (64.8–79.0)	83.5 (77.3–91.5)	79.5 (74.3–86.8)
**Smoking status**						
Current smoker – n (%)	39 (18.3%)	14 (18.4%)	0 (0.0%)	^a^	^a^	21 (21.4%)
Past smoker – n (%)	26 (12.2%)	15 (19.7%)	0 (0.0%)	^a^	^a^	7 (7.1%)
Never smoked – n (%)	61 (28.6%)	20 (26.3%)	^a^	^a^	8 (36.4%)	25 (25.5%)
Unknown – n (%)	87 (40.8%)	27 (35.5%)	^a^	^a^	7 (31.8%)	45 (45.9%)
**Year of RA diagnosis**						
≤2009– n (%)	39 (18.3%)	^a^	^a^	^a^	^a^	18 (18.4%)
2010 – n (%)	45 (21.1%)	16 (21.1%)	^a^	^a^	^a^	22 (22.4%)
2011 – n (%)	66 (31.0%)	17 (22.4%)	^a^	^a^	6 (27.3%)	39 (39.8%)
2012 – n (%)	54 (25.4%)	26 (34.2%)	^a^	^a^	9 (40.9%)	^a^
2013 – n (%)	9 (4.2%)	^a^	0 (0.0%)	^a^	0 (0.0%)	^a^
**RA duration at index date**						
Mean (SD)	7.0 (7.9)	6.4 (8.1)	8.7 (8.1)	5.4 (6.5)	5.7 (5.4)	7.7 (8.2)
Median (IQR)	3.9 (2.1–8.5)	3.3 (2.1–7.2)	4.6 (4.3–10.5)	2.2 (1.3–6.9)	3.8 (1.9–7.8)	5.1 (2.5–9.0)
**Patient history (5 years prior to index date), n (%)**						
Myocardial infarction	8 (3.8%)	^a^	0 (0.0%)	0 (0.0%)	0 (0.0%)	^a^
Other ischemic CV disease	11 (5.2%)	^a^	0 (0.0%)	0 (0.0%)	^a^	5 (5.1%)
Stroke	5 (2.3%)	^a^	0 (0.0%)	0 (0.0%)	^a^	^a^
Cancer	13 (6.1%)	6 (7.9%)	0 (0.0%)	^a^	^a^	^a^
COPD	10 (4.7%)	^a^	0 (0.0%)	0 (0.0%)	^a^	6 (6.1%)
Hypertension	44 (20.7%)	14 (18.4%)	^a^	^a^	5 (22.7%)	20 (20.4%)
Asthma	30 (14.1%)	11 (14.5%)	^a^	^a^	5 (22.7%)	9 (9.2%)
Interstitial lung disease	13 (6.1%)	7 (9.2%)	^a^	0 (0.0%)	^a^	^a^
Diabetes	15 (7.0%)	8 (10.5%)	0 (0.0%)	0 (0.0%)	^a^	^a^
**ACPA titre (U/ml)**						
n	119	76	^a^	10	22	^a^
Median (IQR)	100.0 (8.2–600.0)	340.0 (100.0–600.0)	100.0 (100.0–300.5)	2.2 (0.3–5.3)	0.0 (0.0–1.0)	51.0 (30.9–123.0)
**RF titre (U/ml)**						
n	180	76	7	10	22	65
Median (IQR)	61.0 (16.9–100.0)	99.5 (52.3–100.0)	0.0 (0.0–11.6)	32.9 (21.8–90.8)	0.0 (0.0–6.8)	66.0 (27.0–100.0)
**DAS28-ESR score**						
Mean (SD)	6.3 (1.0)	6.3 (0.9)	6.9 (0.9)	6.0 (0.9)	6.4 (0.8)	6.2 (1.2)
Median (IQR)	6.3 (5.7–7.0)	6.4 (5.7–7.0)	6.7 (6.1–7.4)	5.4 (5.4–7.0)	6.3 (6.0–6.9)	6.2 (5.4–6.7)
High – n (%)	89 (80.9%)	41 (80.4%)	7 (100.0%)	^a^	17 (94.4%)	22 (75.9%)
Moderate – n (%)	20 (18.2%)	10 (19.6%)	0 (0.0%)	^a^	^a^	6 (20.7%)
Low – n (%)	^a^	0 (0.0%)	0 (0.0%)	0 (0.0%)	0 (0.0%)	^a^
Remission – n (%)	0 (0.0%)	0 (0.0%)	0 (0.0%)	0 (0.0%)	0 (0.0%)	0 (0.0%)
**DAS28-CRP score**						
Mean (SD)	5.5 (1.2)	5.8 (0.8)	6.4 (1.0)	5.3 (1.3)	6.0 (0.9)	5.2 (1.3)
Median (IQR)	5.5 (4.8–6.3)	5.7 (5.2–6.3)	6.0 (5.8–6.6)	5.5 (4.5–6.1)	6.1 (5.5–6.7)	5.1 (4.3–6.3)
High – n (%)	70 (51.9%)	27 (60.0%)	^a^	^a^	7 (70.0%)	29 (41.4%)
Moderate – n (%)	60 (44.4%)	18 (40.0%)	0 (0.0%)	^a^	^a^	36 (51.4%)
Low – n (%)	^a^	0 (0.0%)	0 (0.0%)	0 (0.0%)	0 (0.0%)	^a^
Remission – n (%)	^a^	0 (0.0%)	0 (0.0%)	0 (0.0%)	0 (0.0%)	^a^

*ACPA* Anti-citrullinated protein antibodies, *CV* Cardiovascular, *COPD* Chronic obstructive pulmonary disease, *CRP* C-reactive protein, *CV* Cardiovascular, *DAS28* Disease activity score, *DBP* Diastolic blood pressure, *ESR* Erythrocyte sedimentation rate, *IQR* Interquartile range, *RF* Rheumatoid factor, *SBP* Systolic blood pressure, *SD* Standard deviation

^a^Cells suppressed due to small numbers

**Fig. 1:**
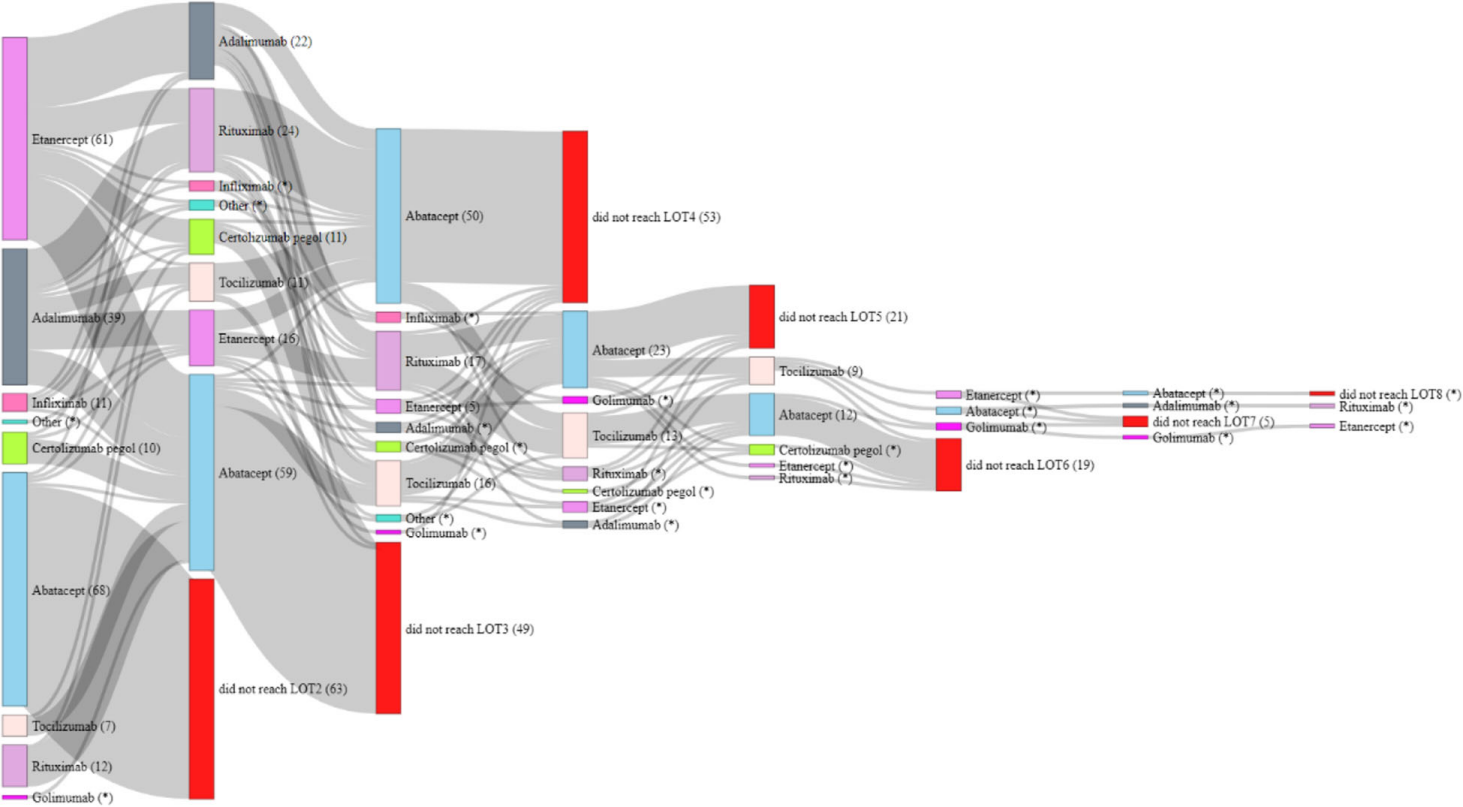
Sankey diagram depicting treatment sequencing for bDMARDs in patients with rheumatoid arthritis. bDMARD: biologic disease modifying antirheumatic drug; LOT: line of therapy

**Fig. 2:**
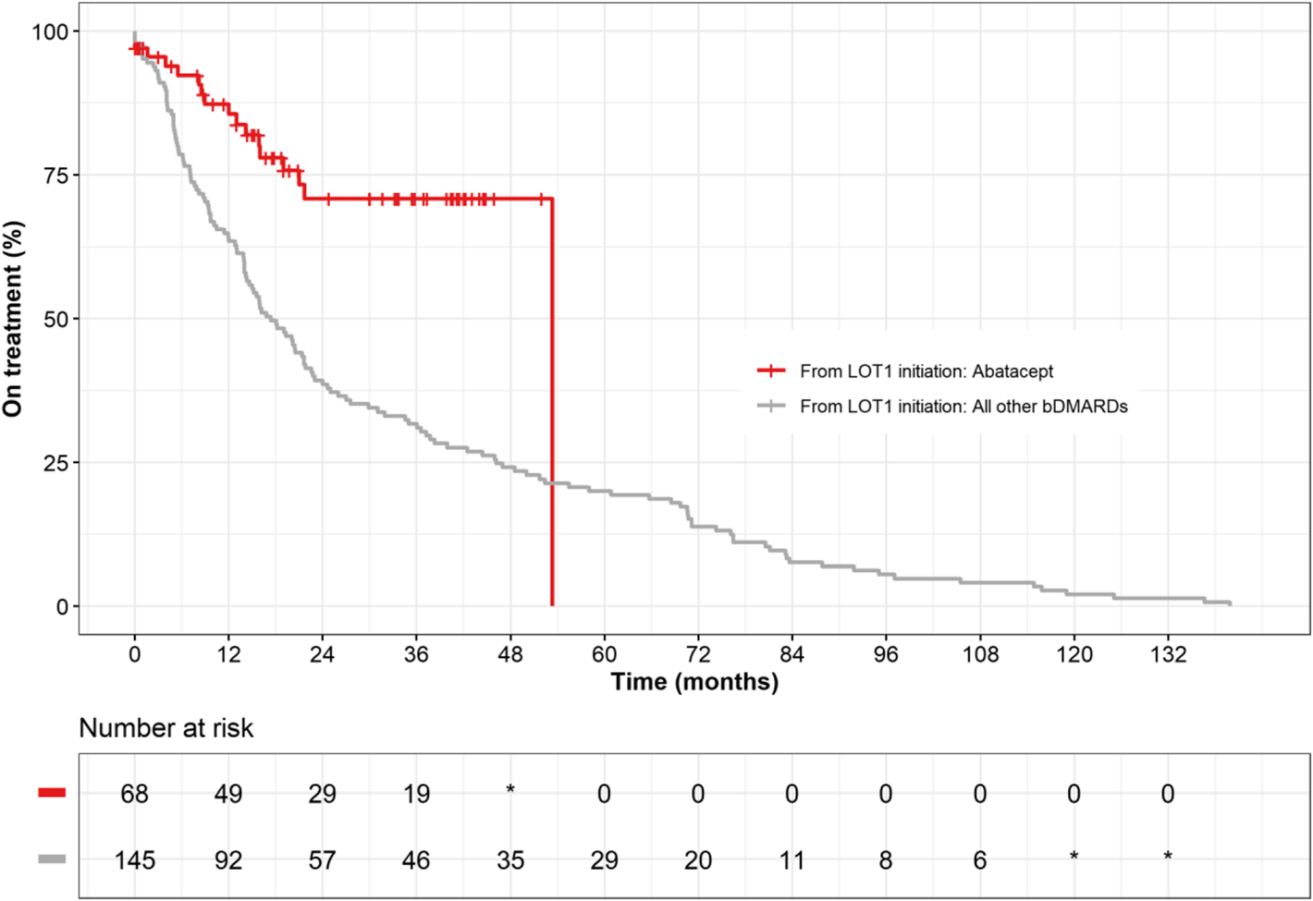
Time on treatment from LOT1 initiation stratified by abatacept vs other bDMARD therapy. Source: Henning et al. [25]

Patients received up to 8 LOTs (Fig. 1). Patients who received abatacept in LOT1 usually did not receive any further lines of treatment (*n* = 63, 92.6%). This was due to patients reaching EOFU (*n* = 51, 81.0%), adverse events (*n* = 5, 7.9%), disease progression (*n* = < 5), or other reasons not otherwise stated (*n* = 5, 7.9%). Patients receiving abatacept at LOT1 (*n* = 68) spent significantly longer on treatment compared with patients receiving other bDMARDs (median 53.4 vs. 17.4 months; *p*< 0.01) (Fig. 2, Table 2). For patients receiving abatacept at LOT1, 85.6, 70.9 and 70.9% of patients were still in receipt of abatacept at 12, 24 and 36 months, respectively, compared with 63.4, 39.3 and 31.7% of patients receiving other bDMARDs, respectively. A similar pattern was observed at LOT2 (*n* = 59), with median time on treatment of 40.1 vs 17.1 (*p*< 0.01) months, respectively.

**Table 2:** Time on treatment from LOT1 initiation, abatacept compared to other bDMARD therapy

bDMARD therapy	Median (months)	R-Mean (months)	Max Follow Up (months)	Month 12 (%)	Month 24 (%)	Month 36 (%)	***P***-value
Abatacept	53.4	41.2	53.4	85.6%	70.9%	70.9%	W:< 0.01* LR:< 0.01*
Other	17.4	31.4	139.9	63.4%	39.3%	31.7%	

*bDMARD* Biologic disease-modifying antirheumatic drug, *LOT* Line of therapy, *LR* Log-Rank, *R-Mean* Restricted-mean (mean survival restricted at maximum follow-up within the cohort), *W* Wilcoxon

* Denotes significance at *p*-value < 0.05

The number of patients who discontinued bDMARD treatment within 6 months and after 6 months of any LOT initiation, stratified by LOT, are summarised in Table 3. The proportion of patients receiving abatacept and still on treatment at EOFU was greater for all LOTs compared with patients who received other bDMARDs. Greater proportions of patients who discontinued other bDMARDs in LOTs 1–4 after 6 months tended to experience infections requiring antibiotics, compared with patients who discontinued abatacept. However, this difference was not statistically significant, which may be due to the low number of recorded infections requiring antibiotics for patients receiving abatacept (Table 4).

**Table 3:** Distribution of patients who discontinued treatment - by LOT and time of discontinuation

LOT	Received treatment, n	Still on treatment at EOFU, n (%)	Discontinued treatment		
			Time to discontinuation, months Mean (SD)	Within 6 months of LOT initiation	After 6 months of LOT initiation
**All patients (*n*** **= 213)**					
LOT1	213	51 (23.9%)	29.4 (31.8)	36 (16.9%)	126 (59.2%)
LOT2	150	42 (28.0%)	20.2 (20.4)	29 (19.3%)	79 (52.7%)
LOT3	101	43 (42.6%)	16.7 (16.2)	20 (19.8%)	38 (37.6%)
LOT4	48	18 (37.5%)	22.2 (25.1)	11 (22.9%)	19 (39.6%)
**Received abatacept**					
LOT1	68	51 (75.0%)	13.0 (12.4)	5 (7.4%)	12 (17.6%)
LOT2	59	40 (67.8%)	12.7 (10.7)	7 (11.9%)	12 (20.3%)
LOT3	50	38 (76.0%)	12.1 (6.9)	4 (8.0%)	8 (16.0%)
LOT4	23	11 (47.8%)	10.7 (11.0)	5 (21.7%)	7 (30.4%)
**Received other bDMARD**					
LOT1	145	0 (0.0%)	31.4 (32.8)	31 (21.4%)	114 (78.6%)
LOT2	91	2 (2.2%)	21.8 (21.6)	22 (24.2%)	67 (73.6%)
LOT3	51	5 (9.8%)	17.9 (17.7)	16 (31.4%)	30 (58.8%)
LOT4	25	7 (28.0%)	29.9 (29.0)	6 (24.0%)	12 (48.0%)

*bDMARD* Biologic disease-modifying antirheumatic drugs, *EOFU* End of follow-up, *LOT* Line of therapy. lOTs 5–8 removed due to a high proportion requiring small number suppression

**Table 4:** Infections requiring antibiotics for patients who discontinued treatment after 6 months of LOT initiation

Variable	LOT1 (***n*** = 126)		LOT2 (***n*** = 79)		LOT3 (***n*** = 38)		LOT4 (***n*** = 19)	
	Abatacept (***n*** = 12)	Other bDMARD (***n*** = 114)	Abatacept (***n*** = 12)	Other bDMARD (***n*** = 67)	Abatacept (***n*** = 8)	Other bDMARD (***n*** = 30)	Abatacept (***n*** = 7)	Other bDMARD (***n*** = 12)
**Infections (requiring antibiotics)**								
Experienced event (%)	^a^	27 (23.7%)	^a^	18 (26.9%)	^a^	13 (43.3%)	^a^	5 (41.7%)
Discontinued treatment (%)	^a^	27 (23.7%)	^a^	18 (26.9%)	0 (0.0%)	12 (40.0%)	^a^	5 (41.7%)

*LOT* Line of therapy

^a^Cells suppressed due to small numbers

**Fig. 3:**
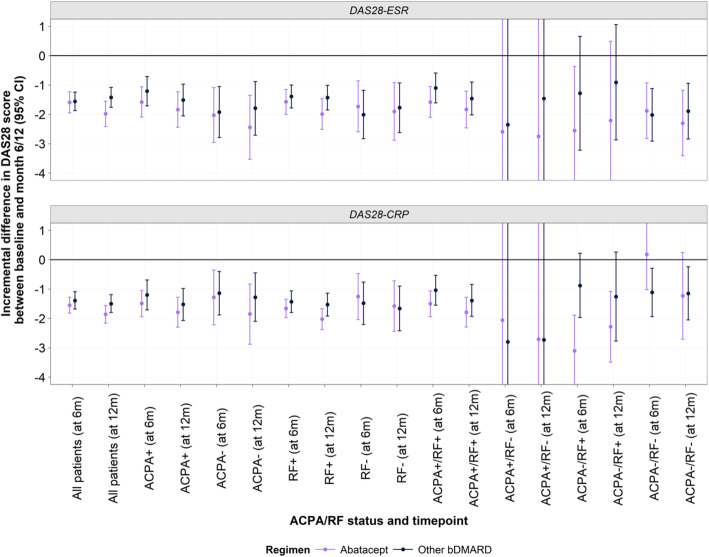
Difference in DAS28-ESR and -CRP scores at 6 and 12 months after LOT initiation. ACPA: anti-citrullinated protein antibodies; bDMARD: biologic disease modifying antirheumatic drug; CI: confidence interval; CRP: C-reactive protein; DAS: disease activity score; ESR: erythrocyte sedimentation rate; LOT: line of therapy; RF: rheumatoid factor. Note: Where applicable, error bars have been capped from −4 to 1 for presentability

Overall, treatment with abatacept or any other bDMARD was associated with reductions in DAS28-ESR and DAS28-CRP scores at 6 and 12 months after any LOT initiation, when adjusted for age and sex (Fig. 3, Tables 5 and 6). At 6 months, there was a greater incremental difference in DAS28-ESR scores between patients receiving abatacept versus other bDMARDs (observed for all ACPA and RF stratifications, with the exception of RF- and ACPA−/RF- subgroups). For DAS28-CRP, there was also a greater incremental difference in scores between patients receiving abatacept versus other bDMARDs (with the exception of RF- and ACPA+/RF- subgroups). Statistical significance was observed for the ACPA−/RF+ subgroup (LG mean: -2.22; 95% CI: − 3.64,-0.81; *p*=0.01). At 12 months, similar trends were observed for DAS28-ESR and DAS28-CRP scores, with statistical significance observed for the overall cohort (LS mean: -0.56; 95% CI: − 1.04,-0.07; *p*=0.03) and the RF+ subgroup (LS mean: -0.49; 95% CI: − 0.95,-0.03; *p*=0.04), respectively.

**Table 5:** Incremental change in DAS28-ESR and DAS28-CRP at 6 months after LOT initiation

Antibody Status	From 6 months								
	Abatacept			Other bDMARD			Incremental difference		
	Cohort (n)	Mean	95% CI	Cohort (n)	Mean	95% CI	LS Mean	95% CI	***P*** value
**DAS28-ESR**									
All patients	92	−1.59	−1.95,-1.23	145	−1.56	− 1.87,-1.24	− 0.04	− 0.45,0.38	0.86
ACPA+	48	−1.58	−2.09,-1.06	60	− 1.21	−1.71,-0.71	−0.37	− 0.97,0.24	0.24
ACPA-	20	−2.03	−2.96,-1.09	30	−1.92	−2.79,-1.05	− 0.11	− 0.94,0.73	0.81
RF+	65	−1.57	−2.00,-1.15	94	−1.39	− 1.78,-1.00	−0.19	−0.69,0.32	0.47
RF-	21	−1.73	−2.59,-0.86	34	−2.01	− 2.83,-1.18	0.28	−0.51,1.08	0.49
ACPA+/RF+	45	−1.58	−2.10,-1.05	47	− 1.10	− 1.61,-0.59	−0.48	− 1.11,0.15	0.14
ACPA+/RF-	^a^	−2.59	−36.06,30.88	^a^	−2.35	− 54.75,50.05	−0.24	−5.27,4.79	0.93
ACPA−/RF+	^a^	−2.55	−4.73,-0.37	6	−1.28	−3.22,0.66	− 1.27	− 3.14,0.60	0.22
ACPA−/RF-	16	−1.88	−2.82,-0.93	24	−2.02	−2.91,-1.12	0.14	−0.76,1.04	0.76
**DAS28-CRP**									
All patients	116	−1.55	− 1.82,-1.28	106	−1.39	− 1.68,-1.09	−0.16	− 0.50,0.18	0.36
ACPA+	48	−1.49	− 1.94,-1.05	42	−1.20	− 1.71,-0.69	−0.29	− 0.83,0.24	0.28
ACPA-	11	−1.28	−2.22,-0.35	21	−1.14	−1.88,-0.40	−0.14	− 1.22,0.93	0.80
RF+	87	−1.66	−1.97,-1.35	69	−1.43	− 1.80,-1.06	− 0.23	− 0.64,0.18	0.27
RF-	17	−1.25	−2.04,-0.47	25	−1.48	−2.21,-0.76	0.23	−0.67,1.13	0.62
ACPA+/RF+	44	−1.50	−1.94,-1.06	38	−1.04	− 1.55,-0.53	−0.46	−1.02,0.11	0.12
ACPA+/RF-	^a^	−2.06	−13.11,9.00	^a^	−2.80	−19.06,13.46	0.74	−2.47,3.96	0.69
ACPA−/RF+	^a^	−3.10	−4.32,-1.89	6	−0.88	−1.97,0.22	−2.22	− 3.64,-0.81	0.01
ACPA−/RF-	6	0.18	−1.02,1.38	15	−1.11	−1.94,-0.29	1.30	−0.08,2.67	0.07

*bDMARD* Biologic disease-modifying antirheumatic drugs, *CI* Confidence interval, *CRP* c-reactive protein, *DAS* Disease activity score, *ESR* Erythrocyte sedimentation rate; n is the number of patients with a DAS score recorded at LOT initiation and at 6 months

^a^Cells suppressed due to small numbers

**Table 6:** Incremental change in DAS28-ESR and DAS28-CRP at 12 months after LOT initiation

Antibody Status	From 12 months								
	Abatacept			Other bDMARD			Incremental difference		
	Cohort (n)	Mean	95% CI	Cohort (n)	Mean	95% CI	LS Mean	95% CI	P value
**DAS28-ESR**									
All patients	61	−1.98	−2.41,-1.55	113	−1.42	− 1.76,-1.08	− 0.56	− 1.04,-0.07	0.03
ACPA+	33	−1.84	−2.44,-1.23	46	−1.51	−2.05,-0.97	− 0.33	− 1.04,0.38	0.37
ACPA-	11	−2.44	− 3.53,-1.35	24	−1.79	−2.71,-0.88	−0.64	− 1.70,0.41	0.23
RF+	42	−1.99	−2.51,-1.46	74	−1.43	−1.85,-1.01	−0.56	− 1.16,0.05	0.07
RF-	14	−1.90	−2.88,-0.92	28	−1.77	−2.62,-0.93	−0.12	− 1.04,0.79	0.80
ACPA+/RF+	31	−1.83	−2.46,-1.21	42	−1.46	−2.02,-0.90	−0.37	−1.12,0.38	0.33
ACPA+/RF-	^a^	−2.75	−28.19,22.70	^a^	−1.46	−59.19,56.28	−1.29	−6.91,4.33	0.67
ACPA−/RF+	^a^	−2.21	−4.91,0.49	6	−0.91	−2.87,1.06	−1.30	−3.75,1.14	0.32
ACPA−/RF-	9	−2.30	−3.41,-1.18	18	−1.89	−2.84,-0.94	−0.41	− 1.55,0.74	0.49
**DAS28-CRP**									
All patients	91	−1.86	−2.16,-1.56	90	−1.50	− 1.80,-1.19	−0.37	− 0.75,0.01	0.06
ACPA+	35	−1.79	−2.30,-1.28	33	−1.52	−2.07,-0.98	−0.26	−0.88,0.35	0.40
ACPA-	9	− 1.85	−2.88,-0.83	16	−1.28	−2.10,-0.45	−0.58	− 1.79,0.64	0.36
RF+	65	−2.02	− 2.38,-1.67	57	−1.53	−1.92,-1.14	−0.49	−0.95,-0.03	0.04
RF-	14	−1.58	−2.44,-0.72	21	−1.66	−2.42,−0.90	0.08	-0.90,1.07	0.87
ACPA+/RF+	33	−1.79	−2.29,-1.28	31	−1.39	− 1.93,-0.84	− 0.40	− 1.03,0.24	0.22
ACPA+/RF-	^a^	−2.71	−13.76,8.35	^a^	−2.73	−15.73,10.28	0.02	−3.62,3.66	0.99
ACPA−/RF+	^a^	−2.28	− 3.49,-1.08	^a^	−1.26	−2.77,0.26	−1.03	− 2.60,0.55	0.23
ACPA−/RF-	^a^	−1.23	−2.71,0.25	12	−1.15	−2.05,-0.24	−0.08	−1.74,1.57	0.92

*bDMARD* Biologic disease-modifying antirheumatic drugs, *CI* Confidence interval, *CRP* c-reactive protein, *DAS* Disease activity score, *ESR* Erythrocyte sedimentation rate, *n* Is the number of patients with a DAS score recorded at LOT initiation and at 12 months

^a^Cells suppressed due to small numbers

The distribution of EULAR responses (good/moderate/no response) tended to be more favourable for patients receiving abatacept than when receiving other bDMARDs at any LOT (22.8%/41.3%/35.9% versus 16.6%/41.4%/42.1% at 6 months, and 27.9%/36.1%/36.1% versus 21.2%/34.5%/44.2% at 12 months) (see Additional file 1: Table 1).

## Discussion

This retrospective chart review study included data from 213 RA patients treated with abatacept at any LOT across four NHS centres in the UK from 2013 to 2017. The mean age of included patients was 55.2 years and patients were predominantly female (71.4%). This aligns with the characteristics of the overall UK RA population, with more women affected by RA than men and diagnosis typically occurring between 40 and 60 years of age [7]. In this study, the median (IQR) duration of RA before starting bDMARD therapy was 3.9 (2.1–8.5) years. Disease duration and number of prior DMARDs can affect treatment response in patients with established RA [26], and delayed start of disease-modifying therapy is associated with reduced disease control and poorer long-term outcomes [27, 28].

Of those patients with known ACPA and RF status (*n* = 115), 66.1% were ACPA+/RF+, 19.1% were ACPA−/RF-, 8.7% were ACPA−/RF+, and 6.1% were ACPA+/RF-. However, there was a large proportion of patients with missing ACPA and RF data (*n* = 94/213 and *n* = 33/213, respectively). This may be explained by the fact that ACPA was only introduced into the updated ACR/EULAR Rheumatoid Arthritis Classification Criteria in 2010 [29] but the majority of patients in this study (70.4%) were diagnosed with RA prior to 2009. It should also be noted that investigations of ACPA and RF are also recommended by NICE [30].

Changes in DAS28 scores observed in this study exceeded the 1.2- threshold considered to be clinically significant changes over time in patients with RA [31, 32]. In particular, RF negative status was associated with a trend towards lower incremental differences in DAS28 score when receiving abatacept. Published literature indicates that both RF and ACPA positivity are important predictors of remission outcomes. For abatacept, ACPA positive status has been associated with better clinical response independent from disease activity [33, 34]. The distribution of EULAR responses further supports the observation of greater improvement in DAS28-ESR scores in patients whilst receiving abatacept.

Patients receiving abatacept spent significantly longer on treatment than patients receiving other bDMARDs (median 53.4 months compared with 17.4 months from LOT1 initiation, respectively, *p*< 0.01). A similar pattern was observed at LOT2, with median time on treatment of 40.1 vs 17.1 months, respectively. This observation has also been reported in other studies where treatment persistence and durability were highest for abatacept initiators. In other real-world studies, abatacept retention was high, particularly when abatacept was used in earlier lines of treatment [24]. Use of abatacept has also been found to impact the overall pattern of ACPA fine specificities over time [35]. This longer duration of treatment adherence is both beneficial in terms of clinical effectiveness but also potentially for patients’ quality of life and that of their family and carers [8, 9].

In both clinical trials and observational studies, abatacept was associated with a lower risk of hospitalised infection compared with other bDMARDs [36, 37]. Indeed, an observational study of abatacept compared with other bDMARDs reported a significant reduction in infections requiring hospitalisation in patients receiving abatacept (HR: 0.37 (95% CI: 0.18–0.75) [38]. In the head-to-head Abatacept versus Adalimumab Comparison in Biologic-Naive RA Subjects with Background Methotrexate (AMPLE) phase 3b trial, serious infections were experienced in 12 (3.8%) and 19 (5.8%) patients, for abatacept versus adalimumab respectively; the majority of these infections resulted in hospitalisation (12 and 18) [39]. In this study, a greater proportion of patients receiving other bDMARDs in LOTs 1–4 experienced infection requiring antibiotics compared with those receiving abatacept when discontinuation was stratified by therapy (i.e. abatacept or other bDMARDs), specifically patients who have discontinued after 6 months from treatment initiation. These findings may indicate that treatment with abatacept is potentially associated with lower rates of infections given that there should be no difference in the recording of antibiotic use by type of bDMARD therapy. Routine use of biologic therapies for RA that are associated with a lower risk of infections requiring antibiotics may benefit the implementation and success of national antimicrobial resistance (AMR) strategies; AMR is a complex and significant public health concern and existing national action plans aim to reduce the need for and use of antimicrobials [40]. However, the apparent association between infections requiring antibiotics and bDMARDS requires further investigation with larger study samples and more recent practice may account for data from the wider RA population.

There are several methodological limitations in this study. As the study was a retrospective chart review, it may have been subject to incomplete or inaccurate original data entry in the medical records, as well as similar issues during study data collection using the eCRF. A further limitation of this study is the extent of missing data (see Additional file 1: Tables 2 and 3). Whilst changes to clinical practice may account for some missing data (e.g. APCA status), incomplete eCRFs also restricted some subgroup analyses. The study was limited to four UK study centres with limited geographical spread, and thus the applicability of the findings to the wider UK population is unknown. The sample size for this study is also relatively modest, as abatacept has a relatively small secondary care market share across rheumatology specialities [41]. This is partly due to the specific clinical recommendations for use of abatacept in the UK [42, 43], as well as NHS England’s commissioning framework which aims to initiate 90% of new patients on the best value biological medicine within 3 months of a biosimilar medicine being launched [44]. However, abatacept is considered to be cost-effective for patients with poor prognosis, with clear benefits in health-related quality of life [45]. In addition, there is a channelling bias associated with the study due to the preferential prescribing of abatacept, partly due to its favourable safety profile. The design of this study may have limited the analysis of treatment discontinuation and pathways. For example, in Fig. 3, discontinuation events whilst receiving abatacept were clustered within the first 24 months following treatment initiation, whereas discontinuation events were spread across the follow-up period whilst receiving other bDMARDs; this is also represented in Table 4. This suggests that if patients can tolerate abatacept in the short-term, their longer-term tolerability was also positive. However, as patients were only eligible for study inclusion if they received abatacept during the study period, any prior bDMARD therapies received in the lookback period would have had to be discontinued, by definition. This ultimately resulted in patients being more likely to reach EOFU when receiving abatacept. Finally, it should be noted that there was an unequal distribution of patients from each of the four centres due to their capacity; eligible patients were randomised by centre.

## Conclusions

This study found clinical benefits associated with the use of abatacept, related to treatment persistence, durability and risk-benefit profile. These findings, along with the literature discussed, support recommendations in the NHS Long Term Plan to keep patient care close to or at home where possible [46]. At the time of writing, the COVID-19 pandemic is ongoing with unprecedented pressures across the entire health care system. The rheumatology community is working to optimise disease management strategies and transition to telehealth [47]. Early research suggests the risk of serious complications from COVID-19 is not increased for patients treated with bDMARDs or tsDMARDs [48]. However, preventative withdrawal of these treatments, which may occur at the time of COVID-19 symptom onset, should be avoided due to the increased risk of relapse and morbidity [48]. Future research should consider how abatacept and other bDMARDs are used in RA management during and after the pandemic.

## Supplementary Information

**Additional file 1: Table S1.** EULAR response for abatacept vs other bDMARDs at 6 and 12 months after LOT initiation. **Table S2.** Summary of missingness for key baseline demographic and clinical variables at index. **Table S3.** DAS score change from LOT initiation missingness.

## Data Availability

The data that support the findings of this study are available from the study centres, but restrictions apply to the availability of these data, which were used under license for the current study and so are not publicly available. Data are however available from the authors upon reasonable request and with permission of the study centre(s).

## Abbreviations

ACPA: Anti-citrullinated protein-peptide antibodies
ACR: American College of Rheumatology
AMR: Antimicrobial resistance
bDMARD: Biologic disease-modifying antirheumatic drug
COVID-19: Coronavirus Disease 2019 [severe acute respiratory syndrome coronavirus 2 (SARS-CoV-2]
csDMARD: Conventional synthetic disease-modifying antirheumatic drug
DAS28-CRP: Disease activity score for 28 joints – C-reactive protein
DAS28-ESR: Disease activity score for 28 joints - erythrocyte sedimentation rate
eCRF: Electronic case report form
EOFU: End of follow-up
EULAR: European League Against Rheumatism
IQR: Interquartile range
LOT: Line of therapy
NICE: National Institute for Health and Care Excellence
NHS: National Health Service
RA: Rheumatoid arthritis
RF: Rheumatoid factor
TNFi: Tumour necrosis factor inhibitors
tsDMARD: Targeted synthetic disease-modifying antirheumatic drug
UK: United Kingdom
