# Supplementary material for: Outcomes in rheumatoid arthritis patients treated with abatacept: a UK multi-centre observational study
Source: BMC Rheumatol. 2021 Feb 4;5:3. doi: 10.1186/s41927-020-00173-0 (PMC7857859; doi:10.1186/s41927-020-00173-0)
Supplement: Supplementary file 1 — Additional file 1: Table S1. EULAR response for abatacept vs other bDMARDs at 6 and 12 months after LOT initiation. Table S2. Summary of missingness for key baseline demographic and clinical variables at index. Table S3. DAS score change from LOT initiation missingness. [file 41927_2020_173_MOESM1_ESM.docx]

**Additional File 1**

**Table S1: EULAR response for abatacept vs other bDMARDs at 6 and 12 months after LOT initiation**

| **EULAR Response** | **6 months** | | **12 months** | |
| --- | --- | --- | --- | --- |
|  | **Abatacept**  **(n = 92)** | **Other bDMARDs**  **(n = 145)** | **Abatacept**  **(n = 61)** | **Other bDMARDs**  **(n = 113)** |
| Good | 21 (22.8%) | 24 (16.6%) | 17 (27.9%) | 24 (21.2%) |
| Moderate | 38 (41.3%) | 60 (41.4%) | 22 (36.1%) | 39 (34.5%) |
| None | 33 (35.9%) | 61 (42.1%) | 22 (36.1%) | 50 (44.2%) |
| bDMARD: biologic disease-modifying antirheumatic drug; EULAR: European League Against Rheumatism; LOT: line of therapy. Note: n refers to the number of unique LOTs in which a patient has DAS28-ESR collected at both initiation and 6 and/or 12 months (a patient may be included in this analysis multiple times depending on the number of LOTs received and the completeness of measurements within each LOT) | | | | |

**Table S2: Summary of missingness for key baseline demographic and clinical variables at index**

| **Variable** | **Missing data at index date* (n = 213)** |
| --- | --- |
| Weight | 62/213 (29.1%) |
| SBP | 82/213 (38.5%) |
| DBP | 83/213 (39.0%) |
| Number of swollen joints | 40/213 (18.8%) |
| Number of tender joints | 41/213 (19.2%) |
| Patient health assessment | 72/213 (33.8%) |
| Care provider health assessment | 181/213 (85.0%) |
| CRP level | 47/213 (22.1%) |
| ESR | 99/213 (46.5%) |
| DAS-ESR score | 103/213 (48.4%) |
| DAS-CRP score | 78/213 (36.6%) |
| ACPA titre | 94/213 (44.1%) |
| RF titre | 33/213 (15.5%) |
| ACPA: anti-citrullinated protein antibodies; CRP: C-reactive protein; DAS28: disease activity score; DBP: diastolic blood pressure; ESR: erythrocyte sedimentation rate; RF: rheumatoid factor; SBP: systolic blood pressure; *last observation carried forward | |

**Table S3:** **DAS score change from LOT initiation missingness**

| **Month** | **Eligible  for data point, n** | **Missing, n (%)** | |
| --- | --- | --- | --- |
|  |  | **DAS-ESR score  change from LOT initiation** | **DAS-CRP score  change from LOT initiation** |
| 6 months | 494 | 257 (52.0%) | 272 (55.1%) |
| 12 months | 369 | 195 (52.8%) | 189 (51.2%) |
| CRP: C-reactive protein; DAS28: disease activity score; ESR: erythrocyte sedimentation rate; LOT: line of therapy | | | |
